# Spinal Epidural Empyema Associated with Bite Wounds in an Indian Crested Porcupine (*Hystrix indica*)

**DOI:** 10.3390/vetsci13050432

**Published:** 2026-04-28

**Authors:** Avital Levy, Ruthie Shviro, Shira Gonen, Nitzan Adam, David Eshar, Orit Chai, Hagar Merav Shamir

**Affiliations:** 1 Ben Shemen Specialist Referral Center, Ben Shemen Youth Village 7311200, Israel; orit18m@gmail.com; 2 The Israeli Wildlife Hospital, Zoological Center Tel Aviv-Ramat Gan (Safari), P.O. Box 1272, Ramat Gan, Israel; ruthieshviro@gmail.com (R.S.); shiragonenshalom@gmail.com (S.G.); nitzanadam@gmail.com (N.A.); deshar@safari.co.il (D.E.); 3 Koret School of Veterinary Medicine, The Hebrew University of Jerusalem, Herzl 229, P.O. Box 12, Rehovot, Israel; merav.shamir@mail.huji.ac.il

**Keywords:** spinal epidural empyema, bite wounds, *Hystrix indica*, Indian crested porcupine, paraplegia, wildlife medicine

## Abstract

Spinal epidural empyema (SEE) is an uncommon but potentially severe cause of spinal cord compression and neurological dysfunction in veterinary patients. Bite wounds involving the vertebral column may result in deep tissue contamination, paraspinal abscessation, and secondary epidural infection; however, such injuries are poorly described in wildlife species. We report a case of SEE associated with chronically infected bite wounds in an adult Indian crested porcupine (*Hystrix indica*) with paraplegia. Physical and neurological examinations revealed exudative paravertebral wounds, paraplegia with preserved nociception, and findings consistent with a thoracolumbar spinal cord lesion. Survey radiography and contrast myelography demonstrated an extradural compressive lesion at L1–L2. Surgical exploration revealed a purulent tract extending from the skin and paraspinal tissues into the vertebral canal, and a left L1–L2 hemilaminectomy was performed with drainage, debridement, lavage, and Penrose drain placement. *Staphylococcus aureus* was isolated from the abscess, and antimicrobial therapy was adjusted based on susceptibility testing. Postoperative management included physiotherapy and environmental modifications to support ambulation. The porcupine regained ambulation within 4 days after surgery and was released back into the wild approximately 50 days postoperatively with normal gait and tail-rattling behaviour. This case highlights bite-wound-associated SEE as an important differential diagnosis in porcupines presenting with paraplegia and draining paraspinal wounds and suggests that surgical decompression combined with prolonged culture-guided antimicrobial therapy and environmental modifications may result in a favourable outcome.

## Simple Summary

An adult Indian crested porcupine was presented with paraplegia and chronically infected bite wounds in the lumbar paravertebral region. Contrast myelography identified an extradural compressive lesion at L1–L2. Surgical exploration confirmed a purulent tract extending from the skin and paraspinal tissues into the vertebral canal, consistent with spinal epidural empyema. *Staphylococcus aureus* was isolated from the abscess, and treatment included surgical drainage, prolonged antimicrobial therapy, and postoperative rehabilitation with environmental modifications to support ambulation. The porcupine regained the ability to walk within 4 days after surgery and was released back into the wild approximately 50 days later with normal gait. This case shows that spinal epidural empyema should be considered in porcupines with paraplegia, especially when draining tracts or paraspinal wounds are present, and suggests that prompt diagnosis and treatment may result in a favorable outcome.

## 1. Introduction

Spinal injury is a common neurological disorder in small-animal clinical practice, with motor vehicle–related trauma accounting for approximately 90% of cases [1,2,3,4,5]. Bite wounds, falls, and gunshot injuries comprise most of the remaining cases, with bite wounds being the most prevalent of these etiologies [6,7,8,9,10,11,12]. Spinal injuries caused by bite wounds are often more complex than those resulting from other traumatic mechanisms. Teeth can penetrate the skin and deeper tissues, and subsequent motion may tear or avulse musculature and vasculature before impacting the vertebral column. Cutaneous lesions may be minimal; however, once penetration occurs, the associated soft tissues, musculature, and any fractured vertebrae should be considered contaminated. The combination of devitalized tissue, compromised perfusion, and dead space created by the bite provides an ideal environment for bacterial proliferation [11,13,14,15,16]. Spinal epidural empyema, which may develop secondarily in some cases, may arise through direct inoculation and/or contiguous extension from paraspinal soft tissue infection [17,18]. Despite the distinctive nature of this injury which combines wound contamination with spinal cord injury, its clinical features, treatment options and prognosis remain poorly characterized in the veterinary literature [1,2,5,10,19,20].

In free-ranging wildlife, the incidence of bite-wound trauma and bite-associated spinal cord injury remains poorly defined. Like companion animals, wildlife may sustain bites during predation or encounters with wild and domestic canids, resulting in vertebral fracture–luxation, epidural infection, and spinal cord compression. The Indian crested porcupine (*Hystrix indica*), a large rodent native to South Asia and the Middle East, is common in our region and shares habitats with dense populations of wild and domestic canids; however, published clinical reports in this species are scarce [21].

Hospital admission of injured or ill wildlife, including porcupines and porcupettes, to wildlife rescue centers (WRCs) has increased markedly over recent decades, with motor vehicle trauma and bite injuries inflicted by wild canids among the most common presenting complaints. Nevertheless, porcupines frequently die during hospitalization, likely due to the severity of their injuries and the challenges associated with timely diagnosis and appropriate treatment [22].

In this study, we report a case of spinal epidural empyema (SEE) with severe spinal cord dysfunction in an Indian crested porcupine (*Hystrix indica*), associated with chronically infected bite wounds. We describe the clinical presentation, imaging findings, therapeutic considerations, and outcome.

## 2. Case Presentation

A 9.5-kg adult Indian crested porcupine (*Hystrix indica*) was presented to the Israeli Wildlife Hospital for paraplegia. Physical examination identified exudative dorsal skin wounds in the paravertebral lumbar region, including one wound on the left side of the trunk with purulent discharge. Neurological examination revealed paraplegia with preserved nociception in both pelvic limbs and the tail, absence of voluntary pelvic limb and tail movement, and preserved voluntary urination and defecation. Neuroanatomic localization was consistent with a T3–S4 spinal cord lesion.

A complete blood count was not performed at presentation due to temporary technical limitations; however, packed cell volume/total solids (PCV/TS; 45%/6.0 g/dL), peripheral blood smear evaluation, and a full serum biochemistry panel including electrolytes were performed. Blood smear examination revealed leukopenia with neutrophilic toxic changes and increased immature neutrophils (left shift) as well as reactive monocytes. Platelet numbers were considered adequate on smear review, and no morphological abnormalities were noted in red blood cells. Serum biochemistry demonstrated increased amylase activity, a nonspecific finding that raised concern for possible pancreatitis but with no impact on the neurological presentation. Survey radiographs confirmed pregnancy, which was considered an incidental finding. Initial management included intravenous crystalloid therapy, calcium gluconate, antimicrobial treatment with tulathromycin and marbofloxacin, anti-inflammatory therapy with meloxicam, and local wound antisepsis using a 2% chlorhexidine gluconate solution.

Several hours after admission, the porcupine aborted a nonviable fetus. Mild neurological improvement was noted on serial examinations after two days of intensive antimicrobial therapy.

General anesthesia was subsequently performed. Premedication consisted of midazolam (0.3 mg/kg IM), butorphanol (0.3 mg/kg IM), medetomidine (0.03 mg/kg IM), and ketamine (1 mg/kg IM). Anesthesia was induced and maintained with isoflurane in 100% oxygen via endotracheal intubation using a nonrebreathing circuit. Following induction, a more thorough physical examination was possible. The quills were secured with tape, revealing bilateral penetrating wounds with purulent discharge on both sides of the lumbar spine, consistent with bite wounds.

## 3. Diagnostic Imaging

**Figure 1:**
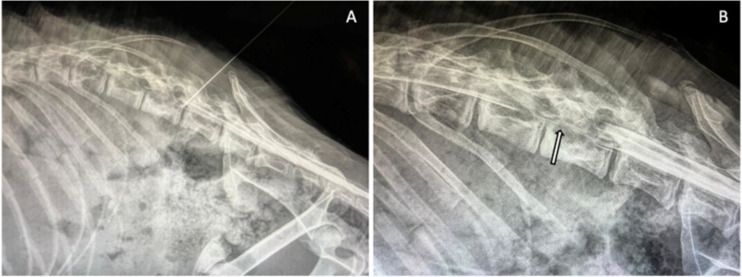
(**A**) Lumbar subarachnoid injection of iohexol at the L2–L3 intervertebral space using a spinal needle for contrast myelography. (**B**) Lumbar myelography, lateral view, showing extradural compression and attenuation of the contrast column at the level of the L1–L2 lumbar vertebra (arrow).

Myelography was performed via a lumbar subarachnoid injection at L2–L3 using iohexol (Omnipaque 300 mg I/mL) administered through a 22-gauge spinal needle (Figure 1). Cerebrospinal fluid (CSF) was collected for routine analysis prior to contrast administration and revealed a normal nucleated cell count (<5 cells/µL) and normal protein concentration (≤25 mg/dL). Iohexol was then injected into the subarachnoid space at a dose of 0.4 mL/kg.

The myelographic study demonstrated attenuation of the contrast column at the level of L1–L2. On the lateral projection, there was dorsal displacement and thinning of the ventral contrast column at L1–L2. On the ventrodorsal projection, medial deviation of the left contrast column was observed at the same level, consistent with a left ventrolateral extradural compressive lesion (Figure 1).

**Figure 2:**
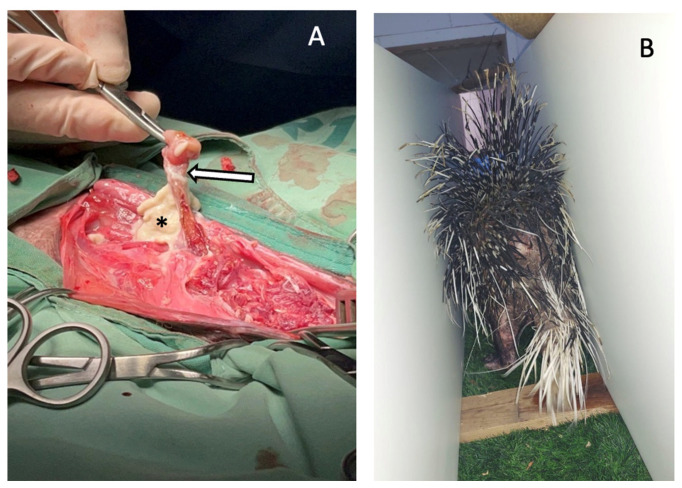
(**A**) Intraoperative view: dense fibrous tissue (arrow) and purulent material (asterisk) identified tracking from the skin through the paravertebral musculature at the level of L1–L2. (**B**) Postoperative rehabilitation setting. Synthetic turf bedding was used to improve limb grip and support ambulation, and a narrow tunnel constructed from parallel cardboard walls was used to encourage forward locomotion while limiting lateral pelvic limb abduction.

The draining tract opening on the skin of the left flank was explored and traced to the level of L2–L3, where a purulent pocket was identified (Figure 2). A left L1–L2 hemilaminectomy allowed further evaluation and confirmed extension of the abscess into the spinal canal. Fibrous tissue and purulent material were removed and the site was thoroughly lavaged with sterile (0.9% NaCl) saline solution. A passive Penrose drain was placed.

## 4. Postoperative Treatment and Outcome

Following surgery, repetitive daily neurological evaluations revealed voluntary movements in the pelvic limbs on postoperative day 1 and return of ambulation by postoperative day 4, although proprioceptive deficits were still present in both pelvic limbs.

*Staphylococcus aureus* was isolated from the abscess following aerobic and anaerobic bacterial culture. Bacterial identification was performed by MALDI-TOF mass spectrometry (Bruker Daltonics, Bremen, Germany), with a species-level identification score of ≥2.0, and antimicrobial susceptibility testing was interpreted according to CLSI standards. Antimicrobial therapy was adjusted to trimethoprim–sulfamethoxazole IM based on susceptibility testing. The isolate was resistant to both marbofloxacin and tulathromycin. CSF was not submitted for microbiological culture, given the unremarkable routine analysis and the limited diagnostic yield of CSF culture reported in veterinary SEE cases [17,23]. Since surgical exploration was performed on the same day, priority was given to culture of purulent material collected directly from the infected site, allowing direct sampling from the epidural/paraspinal lesion. A passive Penrose drain was maintained for 20 days to ensure ongoing drainage of purulent material. At the time of drain removal (performed under sedation), minimal discharge was present and the wounds healed instantly. Trimethoprim–sulfamethoxazole was continued for a total of 60 days.

To facilitate physiotherapy, the bedding was replaced with synthetic turf to improve limb grip and support ambulation (Figure 2). In addition, a simple tunnel was constructed using two parallel cardboard walls, creating a narrow corridor connecting different areas of the enclosure. This design encouraged forward locomotion while limiting lateral pelvic limb abduction (Figure 2). The porcupine had to walk through the tunnel to access food. Further neurological recovery was assessed by daily neurological examinations performed by the attending veterinarians, and continuous video monitoring of the enclosure, which allowed additional assessment of ambulation, appetite, and urination/defecation over time. Once a week the video recordings were reviewed and follow-up neurological examinations were performed by a board-certified veterinary neurologist. Over the first month the porcupine regained stable ambulation and presented the porcupine tail-rattling behavior. The last neurological examination performed 6 weeks post-surgery revealed no neurological abnormalities, normal appetite, and apparent voluntary control of urination and defecation. At this stage the porcupine was released in the same area where it was found 7 weeks earlier. For a free-ranging wild animal, the goal is to return them to the wild without residual neurological deficits and with normal species-appropriate mobility and behavior needed for independent function and survival.

Approximately 50 days postoperatively, the porcupine was transferred to an acclimation enclosure and was subsequently released back into the wild 60 days from presentation.

## 5. Discussion

This report describes spinal epidural empyema (SEE) secondary to a paraspinal abscess associated with bite wounds in an Indian crested porcupine (*Hystrix indica*). Spinal epidural empyema is a severe bacterial infection of the epidural space characterized by accumulation of purulent material within the vertebral canal, leading to progressive myelopathy and potentially severe neurological dysfunction, including paraplegia, as observed in this case [19,20]. Although SEE has been reported in dogs, cats, and humans, to our knowledge it has not previously been described in porcupines [20,23,24,25,26]. Despite the broad regional distribution of *H. indica*, published clinical data on traumatic and infectious spinal disease in this species remain scarce [21].

### 5.1. Diagnostic Considerations

Advanced imaging was not available; therefore, the initial neuroanatomical localization of the lesion relied on neurological findings and was later supported by myelography, which demonstrated an extradural space-occupying lesion compressing the spinal cord at the level of L1 & L2. Survey radiography alone is not the recommended diagnostic modality for SEE, nor can it provide anatomical guidance for surgical exploration. MRI and then CT are both superior for characterizing extradural compressive lesions and delineating the extent of paraspinal soft tissue involvement, discospondylitis, osteomyelitis, vertebral instability, or occult fracture/luxation [17,18,23,25,26].

Myelography was commonly used in the past, but with advanced imaging being more available, MRI should be considered as the imaging modality of choice. This will also reduce the potential risk of iatrogenic leptomeningeal contamination in infectious cases, although no adverse effects were identified in this porcupine. In our case, myelography successfully demonstrated attenuation and deviation of the contrast columns, consistent with a left ventrolateral extradural compressive lesion, that directly continued from the intradermal secretory tract opening to the skin wound on the left side of the trunk. The presence of two penetrating wounds on both sides of the spine with thick purulent discharge draining from the wound on the left, further supported the diagnosis of SEE secondary to penetrating, contaminated bite wounds. The surgical procedure was planned as an exploratory intervention, following the purulent tract from the skin wound into the spinal canal while removing all infected tissues along its course.

The normal CSF findings should also be interpreted cautiously. In dogs and humans with spinal epidural empyema, CSF cytology and protein concentration often reveal mild to moderate neutrophilic pleocytosis, although normal findings have also been reported [17,23,24,25,26,27]. In this porcupine, CSF analysis revealed a normal nucleated cell count and protein concentration despite the presence of an epidural infectious process. This was considered compatible with the final diagnosis because the lesion appeared confined to the epidural space, without clear evidence of substantial subarachnoid or intrathecal involvement. The lack of inflammatory changes in the CSF in this case may also reflect species-related differences or a more acute stage of the disease. Therefore, normal CSF findings should not be interpreted as excluding spinal epidural empyema when other clinical, imaging, and intraoperative findings support the diagnosis.

The lumbar injection site (L2–L3) was selected with consideration of species-specific anatomy [28,29]. In this porcupine, access at L4 was limited by overlap with the sacrum, making L2–L3 the most practical and safe site for subarachnoid contrast administration. This point may be relevant for clinicians performing myelography in hystricomorph rodents and highlights the need to consider anatomic variation when adapting techniques from domestic species.

### 5.2. Treatment Considerations and Outcome

Both conservative treatment with broad-spectrum antibiotics and surgical management involving urgent decompression, drainage, and debridement/lavage have been reported to yield favorable outcomes in dogs and cats, when the appropriate therapy is instituted promptly [15,18,26,30]. In the present case, the lack of advanced imaging also influenced surgical planning, as exploratory decompression had to be planned based on incomplete lesion characterization. Nevertheless, the decision for surgical intervention was dictated by the severity of neurological dysfunction and the presence of extradural spinal cord compression. Exploratory surgery was performed, identifying a purulent epidural collection with a tract extending from the cutaneous draining wound through the paraspinal tissues into the vertebral canal. Surgical management followed principles commonly applied in dogs and cats: hemilaminectomy for extradural decompression, exploration and debridement of the wound tract, copious lavage, placement of a passive drain, and subsequent culture-directed antimicrobial therapy. Bacterial culture and susceptibility testing yielded *Staphylococcus aureus*, consistent with previous reports describing bacterial isolation from porcupine abscesses [31,32]. In published veterinary reports of SEE, the spectrum of causative bacteria has been variable, highlighting the importance of obtaining samples for culture and susceptibility testing whenever possible [17,20,23,26]. Although the optimal duration of antimicrobial therapy has not been definitively established, prolonged treatment courses of 6–16 weeks have been reported [17,20,23,26]. The rapid return to ambulation within four days is consistent with favorable prognostic indicators in domestic species, particularly preserved nociception and timely decompression, and suggests that effective drainage of the epidural and paraspinal infection was a key contributor to neurological recovery.

### 5.3. Species-Specific Context and Knowledge Gaps

Despite the protected status of some porcupine species and growing interest in species conservation, population-level health information in this species remains limited [33,34,35,36].

Similarly, published reference data for hematologic and biochemical parameters in hystricomorph rodents are sparse. Only limited information exists for New World porcupines [33,34], and reference intervals for Old World porcupines, including *Hystrix* spp., are largely unavailable. Establishing species-specific clinicopathologic reference data would improve diagnostic interpretation and may aid prognostication in rescued or hospitalized porcupines.

Overall, this case expands the spectrum of reported causes of severe myelopathy in porcupines and highlights bite-wound-associated SEE as an important differential diagnosis in porcupines presenting with paraplegia, particularly when draining tracts or paraspinal wounds are present. In settings where advanced imaging is unavailable, contrast myelography may provide useful lesion localization; however, definitive diagnosis and treatment may require surgical exploration and drainage combined with prolonged culture-guided antimicrobial therapy.

## 6. Conclusions

This case highlights bite-wound-associated spinal epidural empyema as an important differential diagnosis in porcupines presenting with paraplegia, particularly when draining tracts or paraspinal wounds are present. Prompt recognition and treatment, including surgical exploration, drainage, prolonged culture-guided antimicrobial therapy, and postoperative rehabilitation, may result in a favorable outcome.

## Data Availability

The original contributions presented in this study are included in the article. Further inquiries can be directed to the corresponding author(s).
